# Colony spreading of the gliding bacterium *Flavobacterium johnsoniae* in the absence of the motility adhesin SprB

**DOI:** 10.1038/s41598-020-79762-5

**Published:** 2021-01-13

**Authors:** Keiko Sato, Masami Naya, Yuri Hatano, Yoshio Kondo, Mari Sato, Yuka Narita, Keiji Nagano, Mariko Naito, Koji Nakayama, Chikara Sato

**Affiliations:** 1 Department of Microbiology and Oral Infection, Graduate School of Biomedical Sciences, Nagasaki University, 1-7-1 Sakamoto, Nagasaki, 852-8588 Japan; 2 Health and Medical Research Institute, Advanced Industrial Science and Technology (AIST), Central 6, Higashi 1-1-1, Tsukuba, Ibaraki 305-8566 Japan; 3 Department of Pediatric Dentistry, Graduate School of Biomedical Sciences, Nagasaki University, 1-7-1 Sakamoto, Nagasaki, 852-8588 Japan; 4 Department of Functional Bioscience, Infection Biology, Fukuoka Dental College, 2-15-1 Tamura, Sawara, Fukuoka 814-0913 Japan; 5 Department of Microbiology, Health Sciences University of Hokkaido, 1757 Kanazawa, Tobetsu-cho, Ishikari-gun, Hokkaido 061-0293 Japan

**Keywords:** Microbiology, Molecular biology

## Abstract

Colony spreading of *Flavobacterium johnsoniae* is shown to include gliding motility using the cell surface adhesin SprB, and is drastically affected by agar and glucose concentrations. Wild-type (WT) and Δ*sprB* mutant cells formed nonspreading colonies on soft agar, but spreading dendritic colonies on soft agar containing glucose. In the presence of glucose, an initial cell growth-dependent phase was followed by a secondary SprB-independent, gliding motility-dependent phase. The branching pattern of a Δ*sprB* colony was less complex than the pattern formed by the WT. Mesoscopic and microstructural information was obtained by atmospheric scanning electron microscopy (ASEM) and transmission EM, respectively. In the growth-dependent phase of WT colonies, dendritic tips spread rapidly by the movement of individual cells. In the following SprB-independent phase, leading tips were extended outwards by the movement of dynamic windmill-like rolling centers, and the lipoproteins were expressed more abundantly. Dark spots in WT cells during the growth-dependent spreading phase were not observed in the SprB-independent phase. Various mutations showed that the lipoproteins and the motility machinery were necessary for SprB-independent spreading. Overall, SprB-independent colony spreading is influenced by the lipoproteins, some of which are involved in the gliding machinery, and medium conditions, which together determine the nutrient-seeking behavior.

## Introduction

*Flavobacterium johnsoniae* moves rapidly over solid surfaces by gliding motility, which results in colony spreading. Genetic analyses have shown that the *gld* (gliding) genes *gldA*, *gldB*, *gldD*, and *gldF–N* are required for gliding and that the *spr* (spreading) genes *sprA*–*F* and *sprT* are involved in gliding and in colony spreading, although cells of *F. johnsoniae* mutants with defects in these genes exhibit some gliding movements^1–7^. The GldK–O, SprA, SprE, and SprT proteins are components of the type IX secretion system (T9SS), which is an outer membrane translocon shared by many species in the *Bacteroidetes* phylum^7–10^.

Using the T9SS, *F. johnsoniae* secretes the 669-kDa cell surface adhesin SprB, which is part of the gliding motility machinery used by *F. johnsoniae* cells to move over solid surfaces, and is propelled along a closed helical loop track on the cell body^11–13^. RemA is also part of the gliding machinery because it is known to allow some gliding in the absence of SprB^14^. Gliding was studied in *F. johnsoniae* spreading colonies on nutrient-poor 1% agar PY2 (Peptone-Yeast Extract) medium (1% A-PY2); an Δ*sprB* mutant formed nonspreading colonies on this medium^11^.

Many components of the *F. johnsoniae* T9SS and of the gliding motility apparatus are lipoproteins, including GldB, GldD, GldH, GldI, GldJ, GldK and SprE. In Gram-negative bacteria, lipoproteins are sorted and localized by the lipoprotein localization machinery (Lol), which consists of an ATP-binding cassette transporter (LolCDE), an outer membrane receptor (LolB), and a lipoprotein-specific molecular chaperone (LolA). The completely sequenced genome of *F. johnsoniae* revealed the presence of two genes encoding a LolA homolog; genes encoding LolB or LolCDE homologs were not detected. It was previously reported that a mutant of the *fjoh_2111* gene, which encodes an ortholog of *lolA*, formed nonspreading colonies^15^. The *F. johnsoniae* chromosome contains 257 lipoprotein genes, and most of their functions remain unknown.

Colony spreading of *F. johnsoniae* is influenced not only by cell components, such as SprB, but also by environmental factors, such as the temperature, moisture and nutrient supply. When *F. johnsoniae* cells are cultured on 1% A-PY2, the colony spreads, forming a thin round film^16^. However, colony spreading is prevented by the presence of sugar, including glucose and sugar derivatives, in a dose-dependent manner^17,18^.

Similar colony spreading has been observed for other bacteria, such as *Bacillus subtilis* and *Pseudomonas aeruginosa*. The formation of dendritic patterns is influenced by nutrient and agar concentrations^19^. *P. aeruginosa* spreads and forms dendritic colonies by swarming motility, which is achieved using flagella^20^. The swarming motility changes in response to environmental conditions. For example, tendril formation by *P. aeruginosa* colonies is influenced by the concentration of dirhamnolipids (di-RLs) and 3-(3-hydroxyalkanoyloxy) alkanoic acids (HAAs) in the agar medium^21^. The swarming motility of *P. aeruginosa* is regulated by a two-component signal transduction system comprised of a histidine kinase sensor and a response regulator, like other bacteria^22^.

In the first part of this study, we used Epon-embedding and TEM to examine the SprB-dependent colony spreading of *F. johnsoniae* cells^23^. This revealed that the cells were buried in a matrix containing a thick filamentous network suggesting biofilm formation, and vesicles^23^. Time-lapse fluorescence microscopy and negative stain TEM indicated that a small cell clusters were followed by single cells, and that many cells were connected by filaments at the leading edge of the colony. In the second part of the study reported here, we investigated whether colony spreading of *F. johnsoniae* is affected by agar and glucose concentrations. The motility, population kinetics, matrix production, and cell localization within the biofilm were influenced by the T9SS gliding machinery, agar concentration, and glucose supplementation. Using an *sprB* deletion mutant (Δ*sprB*), we found that SprB was not required for colonies with dendrites to spread on soft agar containing glucose. We employed various deletion mutants to investigate the molecular mechanism of motility adhesin SprB-independent colony spreading. The experiments revealed that SprB-independent colony spreading relies on cell surface lipoproteins.

## Results

### *Flavobacterium johnsoniae* colony spreading is affected by both agar and glucose concentrations

**Figure 1:**
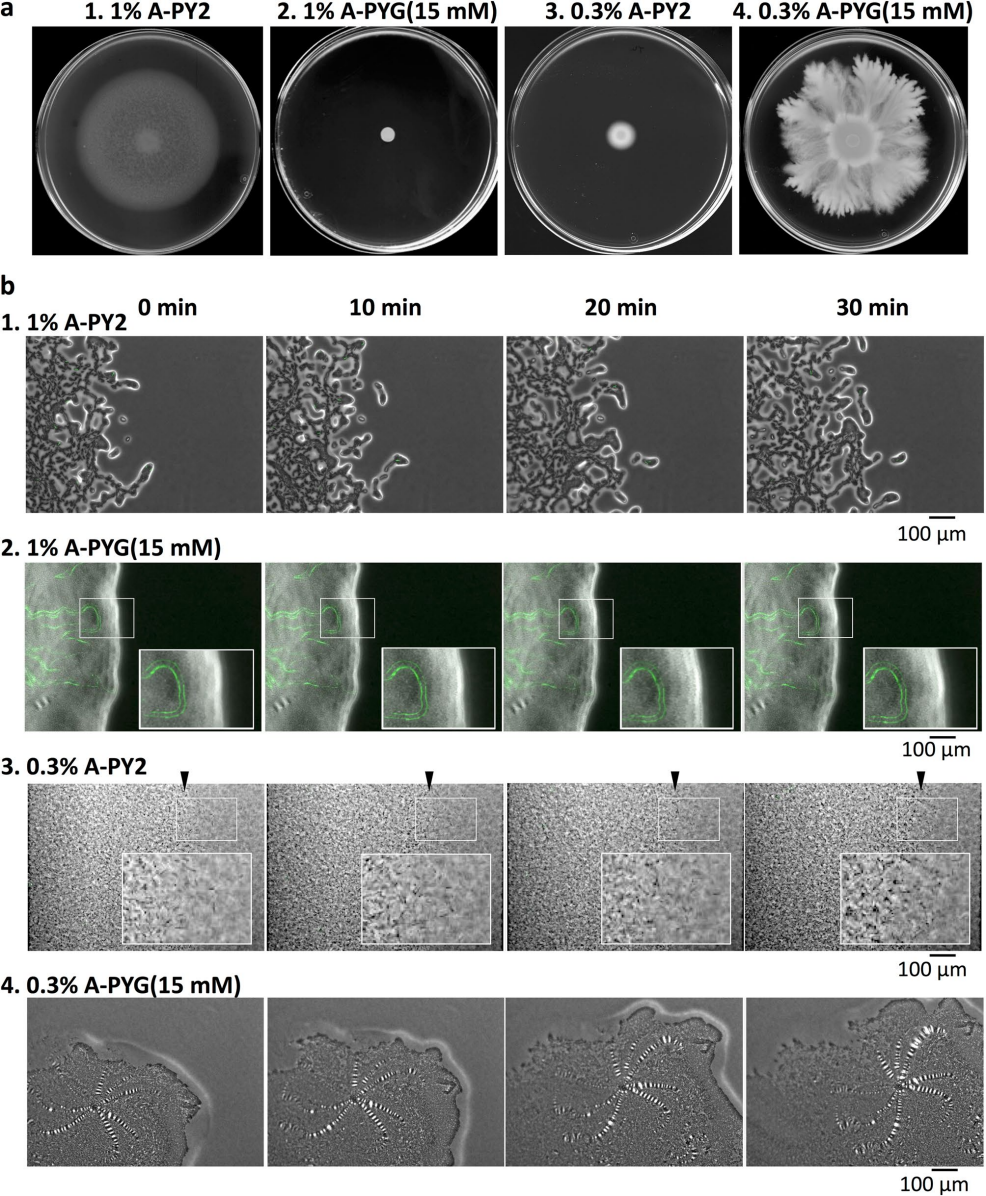
Colony spreading of WT *F. johnsoniae* on media containing different concentrations of agar and glucose. (**a**) Images of a colony spreading in a 9-cm diameter dish (5 days). Panel 1, 1% A-PY2; panel 2, 1% A-PY2 containing 15 mM glucose (1% A-PYG (15 mM)); panel 3, 0.3% A-PY2; panel 4, 0.3% A-PYG (15 mM). Colonies spread well on 1% A-PY2 and 0.3% A-PYG (15 mM). (**b**) Effect of agar and glucose concentrations on the behavior of bacteria in a spreading WT colony. Cells expressing GFP in their cytoplasm were added to the inoculated bacterial solution at a concentration of 1%, and movement of the bacterial cells at the colony edge was monitored. Images were recorded by fluorescence microscopy at 30-s intervals for 30 min. Panel 1, 1% A-PY2; panel 2, 1% A-PYG (15 mM); panel 3, 0.3% A-PY2; panel 4, 0.3% A-PYG (15 mM). At the leading edge of a spreading colony on 1% A-PY2 (panel 1), a small cell cluster moved outward toward the tip of a branch (Fig. S2a). By contrast, in the tip regions at the spreading front on 0.3% A-PYG (15 mM) (panel 4), windmill-like, flat structures including periodic stripes were carried outwards (Fig. S2d1). Time is shown at the top. The subpanels in row 2 and 3 are enlarged images of the regions marked by white rectangles. Arrowheads indicate the position of the leading edge of the colony in row 3. See Fig. S2 for selected frames showing the edge of each bacterial colony during 10 min of video capture.

To examine the effect of the culture medium on colony spreading, a 1-μl drop of washed *F. johnsoniae* wild-type (WT) cells was inoculated on media containing different concentrations of agar, and the cultures were incubated at room temperature (RT) (25–26 °C) for 5 days. On nutrient-poor 1% A-PY2, the cells grew and spread radially from all edges of the inoculated spot at the same speed, forming a large circular colony (Fig. 1a panel 1). On 1% agar PY2 medium supplemented with 15 mM glucose (1% A-PYG (15 mM)), colony spreading was inhibited (Fig. 1a panel 2). The radius of the developing colony on nutrient-poor 1% A-PY2 depended on the incubation time. To investigate the effect of the physical strength of the culture substrate on colony spreading, WT cells were inoculated on media with a lower agar concentration (0.3% agar PY2 (0.3% A-PY2)) (Fig. 1a panel 3). Surprisingly, the *F. johnsoniae* cells formed a circular colony that grew within the small inoculation circle and was only slightly larger than the initial inoculation spot (Fig. 1a panel 3), suggesting that cell mobility was low on the soft substrate. The addition of 15 mM glucose to 0.3% A-PY2 (0.3% A-PYG (15 mM)) induced spreading and dendrite formation (Fig. 1a panel 4), and spreading at the edges was uneven. Further examination of growth conditions, including 0.5% agar and 5 mM glucose, confirmed that colony spreading was influenced by the agar concentration and glucose supplementation (Fig. S1a,b). In the absence of glucose, colony spreading on agar medium decreased as the agar concentration decreased, while in the presence of glucose, colony spreading increased as the concentration of agar decreased. The colonies that spread on agar medium containing glucose were generally dendritic, whereas those formed on medium without glucose were not. To address the mechanism that causes this phenomenon, cell movements in colonies grown on different culture media were next monitored and analyzed.

### Movement of single bacterial cells in a colony in response to different concentrations of agar and glucose in the substrate

1% of the inoculated cells were fluorescently labeled and colony spreading was monitored using time-lapse microscopy (Fig. S2a). On 1% A-PY2, the cells grew and spread radially to form a large circular colony (Fig. 1a panel 1). At the leading edge of the spreading colony, a small cell cluster at the tip of a branch moved outwards, and the cluster was followed by single cells (Figs. 1b panel 1, S2a). Herein, the same experiment was performed on 1% A-PYG (15 mM). The colony did not spread, and long lines of cells producing GFP were apparent within it (Figs. 1b panel 2, S2b). Because *F. johnsoniae* cells divide along a single axis, it is reasonable to propose that the cells in a filament all originate from the same parent cell. Parallel lines of cells sometimes formed, probably due to the division of multiple similarly-orientated cells (Fig. 1b panel 2), suggesting that there was a preferred orientation. The movement of individual cells in the colony was not detected.

On 0.3% A-PY2, each bacterial cell moved back and forth from a point (Figs. 1b panel 3, S2c), and the colony did not expand beyond the circular area inoculated (Figs. 1a panel 3, S2c). By contrast, on 0.3% A-PYG (15 mM) the individual bacterial cells moved in all directions and spread towards the outside of the colony. Unlike cells on 1% A-PY2, the bacterial clusters at the edge of the dendritic colonies did not move along a specific path and lines of cells did not form (Figs. 1b panel 4, S2d1, S2d2). Moreover, in the dendritic tip regions at the spreading front, windmill-like flat structures with periodic stripes comprised of crystal-like bright squares were rotated and moved outwards and away from the inoculation spot as the colony expanded (Figs. 1b panel 4, S2d1, S2d2); these crystal-like structures were not apparent at concave edge regions (depressions) of the dendritic colonies (Fig. S2d3). The windmill-like rolling centers in each leading tip are interpreted as machinery related to dendritic growth.

To study the movements of bacteria around the windmill-like rolling centers, fluorescence labeled *F. johnsoniae* cells were inoculated on 0.3% A-PYG (15 mM), and the top surface of the dendrites formed was monitored using phase contrast microscopy and confocal laser scanning fluorescent microscopy (CLSM) to track the cells. A windmill-like structure was found at one edge of the upper surface of a thin dendrite using phase contrast microscopy (Fig. S2e1 top left). CLSM of the same level, showed that cells were distributed around (Fig. S2e1 bottom left). The orientation of the cells was specific to their neighboring ‘windmill’ arm; each arm was surrounded by or attached to cells oriented in a similar direction to it and to one another.

Next, the same region was inspected for 100 s by time lapse CLSM to study the movements of the cells present. At first sight, the cells of this surface level seemed to be stationary or to oscillate back-and-forth from the points where they were (Fig. S2e1 top right Movie). However, when the cumulative shift of each cell during this period was calculated and depicted by an arrow, most cells did move relative to their neighboring windmill arm by a small but significant amount; cells around the arms at the top and top right in Fig. S2e2, slid largely toward the bottom left, while the cells around the bottom arms just slid slightly to the left. Overall, the windmill-like structure appeared to rotate counterclockwise, and moved towards the bottom left, as in fact observed during the microscopy.

Afterwards, the 3D distribution of cells around and under the windmill-like structure was determined using LCSM (Fig. S2e3 top right Movie). As the depth of focus was increased from the surface, cells were distributed and oriented differently, regardless of the windmill-like structure (Fig. S2e3 lower left, middle and right panels). Overall, the massive movement of the cell population in the dendritic tips suggests gliding of the entire biofilm structure itself.

### Dendritic colony formation by Δ*sprB* cells on 0.3% A-PYG (15 mM)

**Figure 2:**
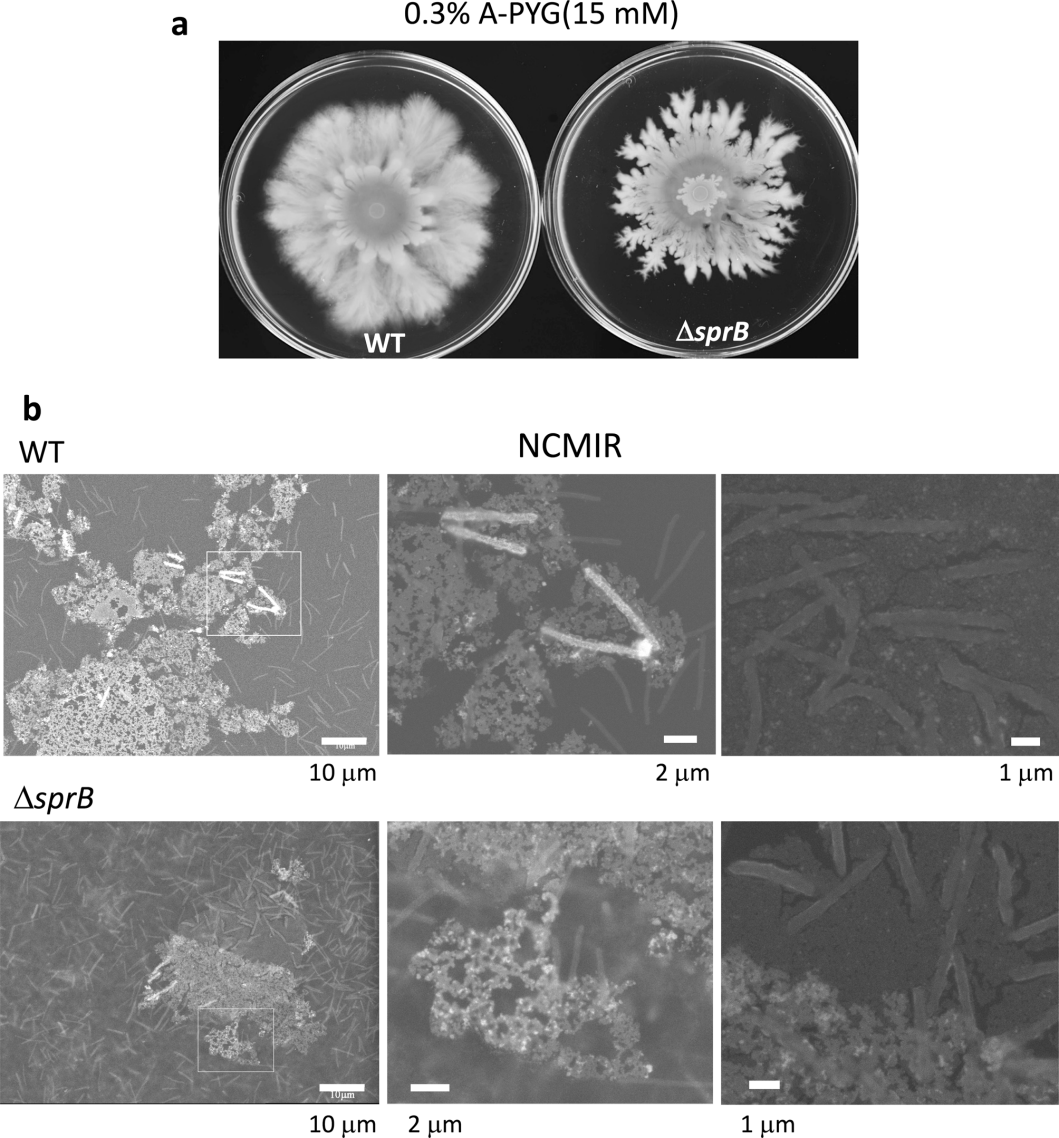
Colony spreading of WT and Δ*sprB* mutant *F. johnsoniae* on 0.3% A-PYG (15 mM). (**a**) Optical microscopy image. The branching pattern of the Δ*sprB* colony was less complex than that of the WT. (**b**) Spreading colonies of *F. johnsoniae* immersed in aqueous glucose buffer imaged by ASEM. The colonies were aldehyde-fixed and stained using the NCMIR method. The top of the biofilm was observed. Both WT and Δ*sprB* mutant cells were buried in the extracellular matrix covering the top of the colony, reflecting biofilm formation. Upper panels, WT; lower panels, Δ*sprB* mutant.

Next, we investigated whether gliding motility involving the adhesin SprB was required for dendritic colony formation in *F. johnsoniae*. To this end, we first evaluated the spreading phenotype of a *F. johnsoniae sprB* mutant (Δ*sprB*). Although the colony spreading of Δ*sprB* was inhibited on 1% A-PY2 and cells only grew within the small inoculation spot^11^, dendritic colonies formed on 0.3% A-PYG (15 mM) (Fig. 2a). The branching pattern of the colony was simpler than the branching pattern formed by the WT (Figs. 2a, S3 Movie). As observed by time-lapse microscopy, colony expansion occurred in two stages: an initial growth-dependent phase followed by a secondary gliding motility-dependent phase (Fig. S3 Movie). During the second phase, the dendritic colony of WT *F. johnsoniae* developed branches that separated further, forming many very fine branches as the colony expanded.

### Dendritic colony formation by different gliding- or spreading- mutant cells on 0.3% A-PYG (15 mM)

T9SS mutant strains of *F. johnsoniae* do not form spreading colonies on 1% A-PY2^10^. To investigate whether T9SS is involved in spreading on 0.3% A-PYG (15 mM), we studied the spreading phenotypes of single-gene mutants of each gene of the T9SS (*gldK–O*, *sprT*, and *sprE*) and of single-gene mutants of each gene of the motility protein (*gldA, gldB, gldD, gldF–J*). All of the mutants exhibited the initial growth-dependent phase, but none exhibited the secondary colony expansion phase dependent on gliding motility (Fig. S4)^4,5,13,15,24,25^. Together, the data show that both the spreading machinery and the gliding machinery, including the T9SS, are essential for the secondary colony expansion phase but not for the initial growth-dependent spreading phase.

### Biofilm formation of *F. johnsoniae* dendritic colonies observed using atmospheric scanning electron microscopy (ASEM)

We employed ASEM to image the structure of dendritic colonies in wet conditions; the method was successfully used earlier to observe biofilms of *Staphylococcus aureus*^21^ and *Propionibacterium acnes*^26^. WT or Δ*sprB F. johnsoniae* colonies were fixed with 3.5% glutaraldehyde buffer and then stained using the National Center for Microscopy and Imaging Research (NCMIR) staining method^27^. Once fully stained, each sample was placed upside down on an ASEM dish (i.e., with the top colony surface in contact with the base of the dish), immersed in aqueous solution and imaged from below through the dish window using the inverted SEM at an acceleration voltage of 30 kV. This allowed a 2–3 μm specimen depth from the top surface of colonies spreading on 0.3% A-PYG (15 mM) to be observed. The experiment revealed that WT and Δ*sprB F. johnsoniae* cells were buried in an extracellular matrix that covered the top of the colony (Fig. 2b), indicating biofilm formation.

### Biofilm formed on 0.3% A-PYG (15 mM) has thick- and thin-filaments and vesicles between the cells in the bottom sides of WT and Δ*sprB* colonies

**Figure 3:**
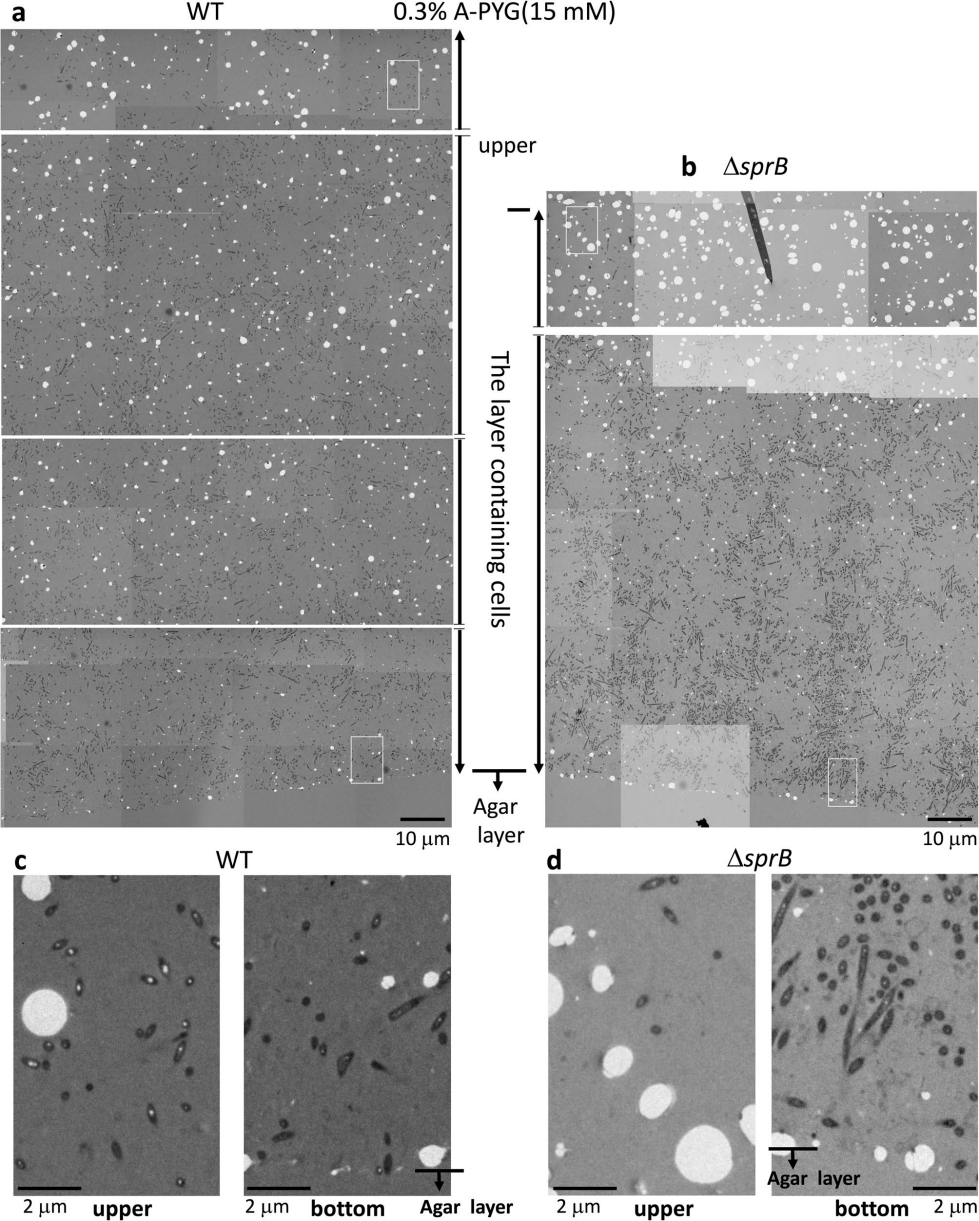
TEM of WT and Δ*sprB* mutant *F. johnsoniae* spreading colonies on 0.3% A-PYG (15 mM). (**a–d**) Colonies were embedded in Epon resin, sliced into 70-nm sections, and observed by TEM. Sections of WT (**a**) and Δ*sprB* (**b**) colonies are shown. The cells were interspersed within the intercellular matrix. (**c,d**) High magnification images of a WT colony (**c**) and a Δ*sprB* colony (**d**). In both cases, the left and right panels are images of the upper and lower regions of the spreading colony, respectively.

**Figure 4:**
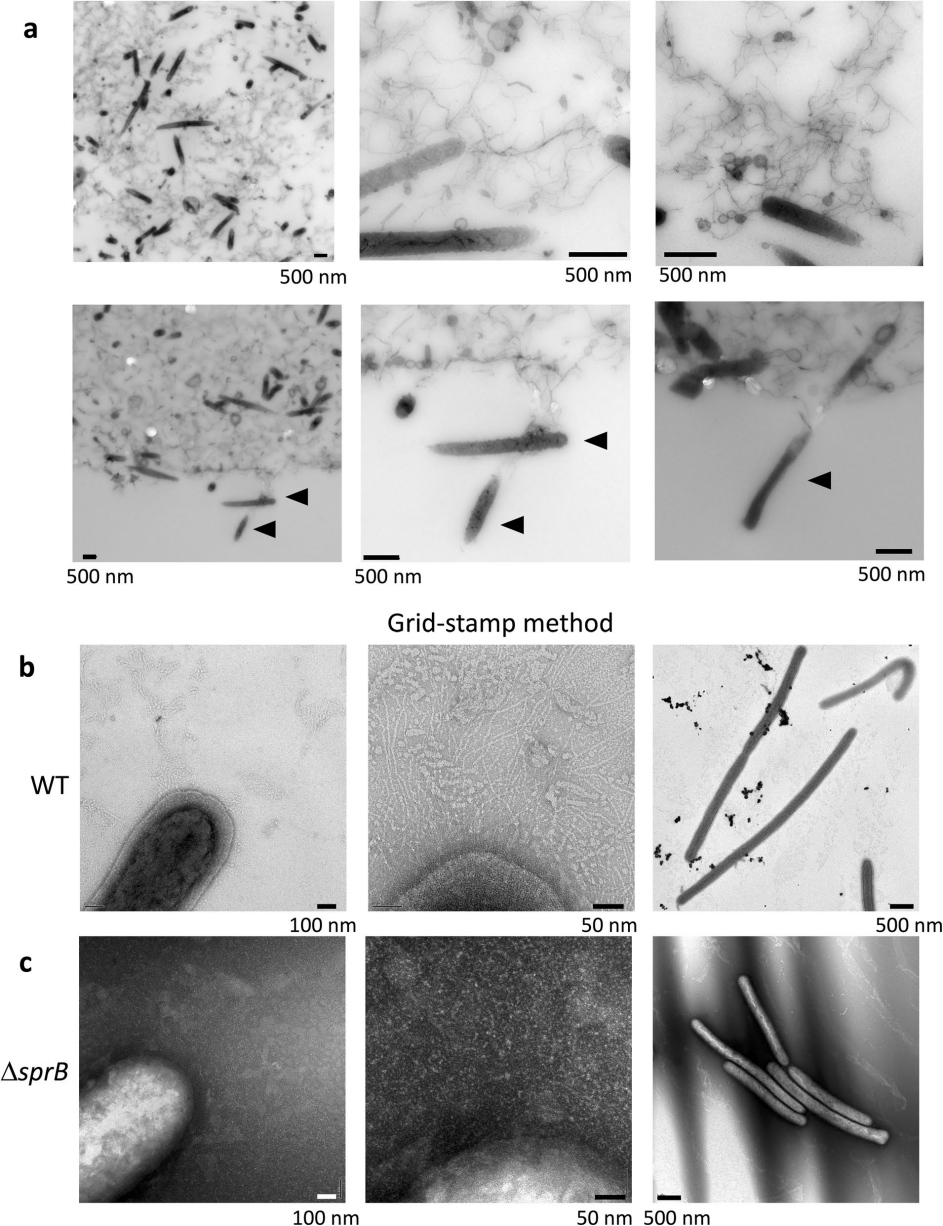
Higher magnification images of the spreading colonies shown in Fig. 3. (**a**) TEM image of a 400 nm-thick, unstained, Epon section of a WT colony spreading on 0.3% A-PYG (15 mM). The space between bacterial cells is occupied by fibers and vesicles (upper panels). Migration of bacterial cells into the 0.3% agar layer (lower panels). WT cells metastasize into the agar medium (arrowhead). (**b,c**) Grid-stamp images of the spreading front region of WT (**b**) and Δ*sprB* (**c**) colonies.

We next used Epon resin-embedded serial thin section (70 nm thickness) and TEM to observe the bottom side of WT colonies on 0.3% A-PYG (15 mM) and to analyze the cells and the intercellular matrix during colony spreading (Fig. 3a). The WT cells were interspersed within the intercellular matrix (Fig. 3c), unlike on 1% A-PY2 where they were concentrated at the bottom^23^. The relatively thick sections (400 nm thickness) allowed the intercellular space to be efficiently visualized, revealing a matrix. Similar to the situation on 1% A-PY2, this matrix contained many thick extracellular fibers that intertwined to form a network and secreted vesicles, further suggesting biofilm formation (Fig. 4a). Furthermore, at the border between the colony and the agar substrate, *F. johnsoniae* WT cells were observed to migrate into the agar layer, reflecting the mobile nature of the strain (Fig. 4a lower row, arrowheads). Furthermore, bacteria were generally distributed at the same cell density throughout the biofilm, from the lower to the upper surface (Fig. 3a,c).

The colony formed by the WT was thicker than the colony formed by the Δ*sprB* mutant (Fig. 3a,b). Δ*sprB* cells were basically distributed throughout the Δ*sprB* colonies, but compared to the WT, they were more densely packed and clustered (Fig. 3b,d), which is attributable to the reduced motility of the mutant. Grid stamp TEM images of the advancing front (top surface) of comparable colonies spreading on 0.3% A-PYG (15 mM) (see “experimental methods” in the first part of this study^23^ for details) revealed many intercellular fibers around WT cells (Fig. 4b, middle panel), whereas fewer fibers were detected around Δ*sprB* cells (Fig. 4c, middle panel). These structural differences in the extracellular matrix might account for the different cell clustering and for the different dendritic branching patterns observed for WT and Δ*sprB* colonies (Fig. 2a). However, inside the dendritic protrusions, the number of fiber structures around Δ*sprB* cells was comparable to the number around WT cells. We conclude that both WT and Δ*sprB F. johnsoniae* formed biofilms on 0.3% A-PYG (15 mM). The dark spots of high electron density observed in the WT cells on 1% A-PY2^28^ were not observed in WT (Fig. 4b right panel) or Δ*sprB* cells on 0.3% A-PYG (15 mM) (Fig. 4c right panel).

### Colony spreading is dependent on the lipoproteins

**Figure 5:**
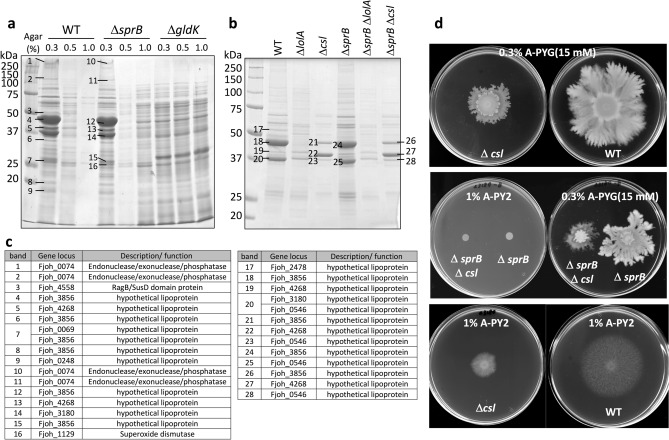
SDS-PAGE analysis of the lipoproteins essential for colony spreading. (**a**) Comparison of the cell surface proteins of WT, Δ*sprB* and *gldK F. johnsoniae* on 0.3, 0.5 and 1% A-PYG (15 mM). Protein bands 1–16 were analyzed by peptide mass fingerprinting. A greater abundance of lipoproteins was present under colony expansion conditions on 0.3% A-PYG (15 mM) than under non-expansion conditions on 1% A-PYG (15 mM). (**b**) Comparison of the cell surface proteins of WT, Δ*lolA*, Δ*csl*, Δ*sprB*, Δ*sprB* Δ*lolA* and Δ*sprB* Δ*csl F. johnsoniae* on 0.3% A-PYG (15 mM). Protein bands 17–28 were analyzed by peptide mass fingerprinting. (**c**) Identification of protein bands present in the SDS-PAGE gel shown in sections a (band nos. 1–16) and b (band nos. 17–28) using mass spectrometry. (**d**) Colony spreading of WT, Δ*csl,* Δ*sprB* and Δ*sprB* Δ*csl F. johnsoniae* on 1% A-PY2 and 0.3% A-PYG (15 mM).

To further identify key proteins necessary for biofilm formation, we compared the expression of cell surface proteins by WT, Δ*sprB* and *gldK F. johnsoniae* colonies on substrates with different agar concentrations. The cell surface proteins were suspended by vortexing the spreading colonies in buffer and analyzed by SDS-PAGE (Fig. 5a). Under colony expansion conditions, the amounts of lipoproteins, including Fjoh_0069, Fjoh_0248, Fjoh_3180, Fjoh_3856 and Fjoh_4268, in the WT colony and in the Δ*sprB* colony on 0.3% A-PYG (15 mM), were more than those on 1% A-PYG (15 mM) (Fig. 5a,c).

Based on the above, we thought that lipoproteins might be involved in colony spreading on 0.3% A-PYG (15 mM). To test this hypothesis, we constructed *Fjoh_0069*, *Fjoh_0248*, *Fjoh_3180*, *Fjoh_3856* and *Fjoh_4268* single gene deletion mutants. The secondary gliding motility-dependent phase of colony spreading was inhibited for Δ*Fjoh_3180* (Fig. 5d). Because of this we suggest calling the *Fjoh_3180 gene csl*, for colony spreading-related lipoprotein. In contrast, the absence of one of the surface proteins Fjoh_0074, Fjoh_0546, Fjoh_2478 and Fjoh_3856, did not affect colony expansion (data not shown).

*Flavobacterium johnsoniae* has many lipoproteins, including GldK, the secretion protein GldJ that is also involved in secretion, and the motility protein GldH. In *F. johnsoniae*, the *Fjoh_2111* gene encodes an ortholog of *lolA*. We examined the expression of the cell surface proteins in mutants lacking *lolA* (Δ*lolA*), *csl* (Δ*csl*), both *lolA* and *sprB* (Δ*lolA* Δ*sprB*) and both *csl* and *sprB* (Δ*csl* Δ*sprB*). Both Δ*lolA* mutants were deficient in outer membrane lipoproteins that were significantly expressed in WT and Δ*sprB* colonies spreading on 0.3% A-PYG (15 mM) (Fig. 5b,c), and further, unlike the WT, both Δ*lolA* mutants formed nonspreading colonies on the agar medium (Fig. S5).

Next, the protein contents of the membrane fractions and the cytoplasmic fractions obtained from Δ*lolA,* Δ*csl* and WT colonies were analyzed. The amount of Fjoh_3856 protein present in membrane fractions from the Δ*lolA* and Δ*csl* mutants was less than in membrane fractions from the WT (Fig. S6 arrow). In contrast, the SDS-PAGE protein patterns of the soluble fraction (cytoplasmic and periplasmic proteins) from these three strains were similar. In addition, colony expansion of the Δ*sprB* Δ*csl* double mutant on 0.3% A-PYG (15 mM) was partially inhibited compared to colony expansion of the Δ*sprB* mutant (Fig. 5d middle right). Colony expansion of the Δ*csl* mutant on 1% A-PY2 was partially suppressed (Fig. 5d bottom left), cells deficient in SprB protein formed rigid colonies (Fig. 5d middle left).

### Biofilm formation by spreading Δ*csl* colonies on 0.3% A-PYG (15 mM)

**Figure 6:**
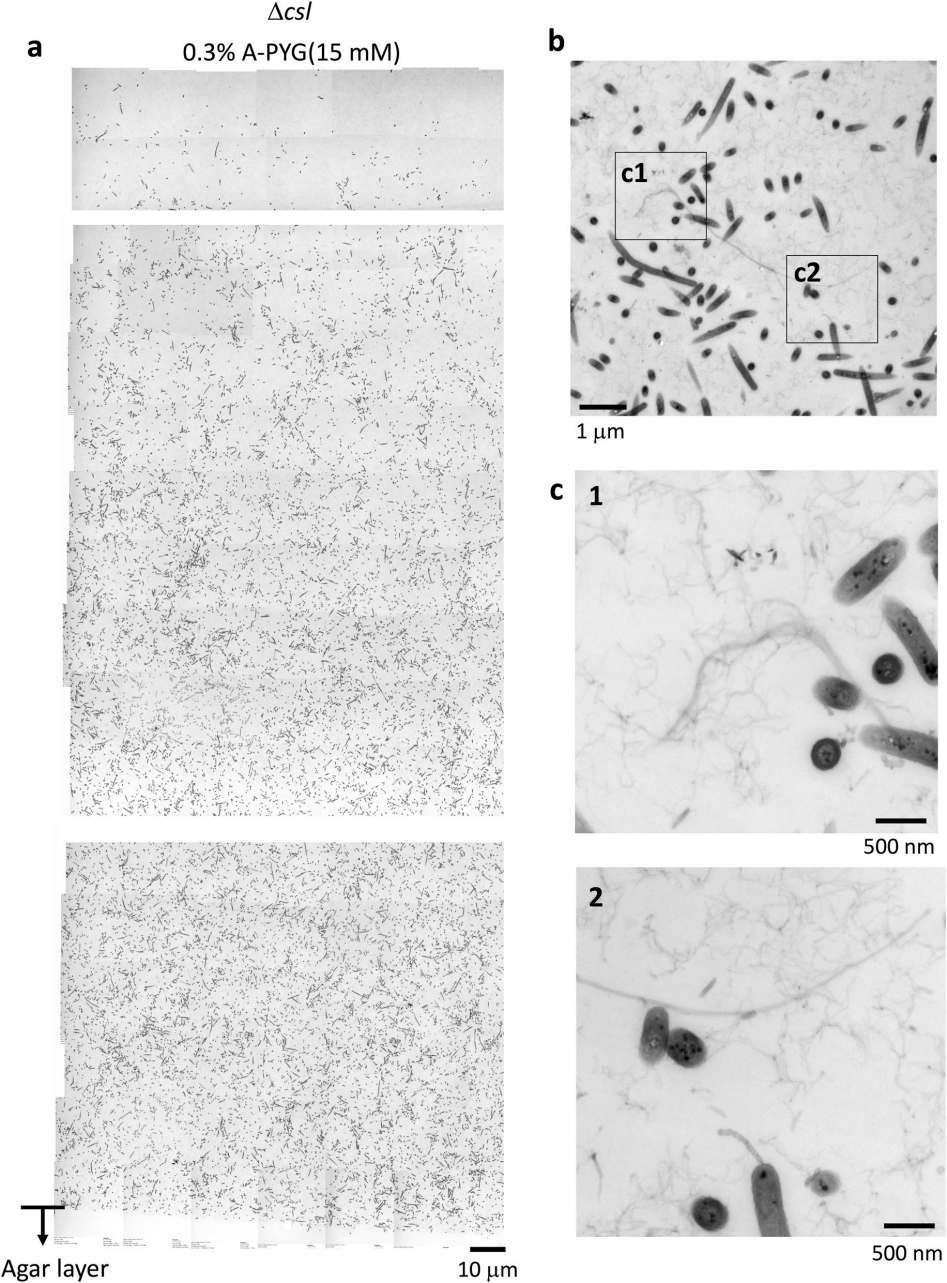
TEM of Δ*csl* mutant *F. johnsoniae* spreading colonies on 0.3% A-PYG (15 mM). Colonies were embedded in Epon resin, sliced into 70-nm serial sections, and observed by TEM. (**a**) Sections of Δ*csl F. johnsoniae* colonies. Similar to the WT and Δ*sprB*, the cells were interspersed within the intercellular matrix. (**b**) TEM image of a 400 nm-thick, unstained, Epon section of a Δ*csl* mutant colony spread on 0.3% A-PYG (15 mM). The intercellular space between bacterial cells was occupied by fibers and vesicles. (**c**) Higher magnification images of the squares in (**b**). Again, similar to WT and Δ*sprB*, the intercellular matrix was occupied by many intertwined fibers and secreted vesicles, suggesting biofilm formation.

To analyze the Δ*csl* mutant cells and the intercellular matrix in a colony (Fig. 5d top left) that spread less than the WT on 0.3% A-PYG (15 mM), we examined Epon resin-embedded thin sections (70-nm thickness) using TEM (Fig. 6a). The space between the bacterial cells appeared to be occupied by many intertwined fibers and by secreted vesicles (Fig. 6b,c), similar to the WT (Figs. 3a, 4a).

### Mutants with reduced lipoprotein export release many vesicles

**Figure 7:**
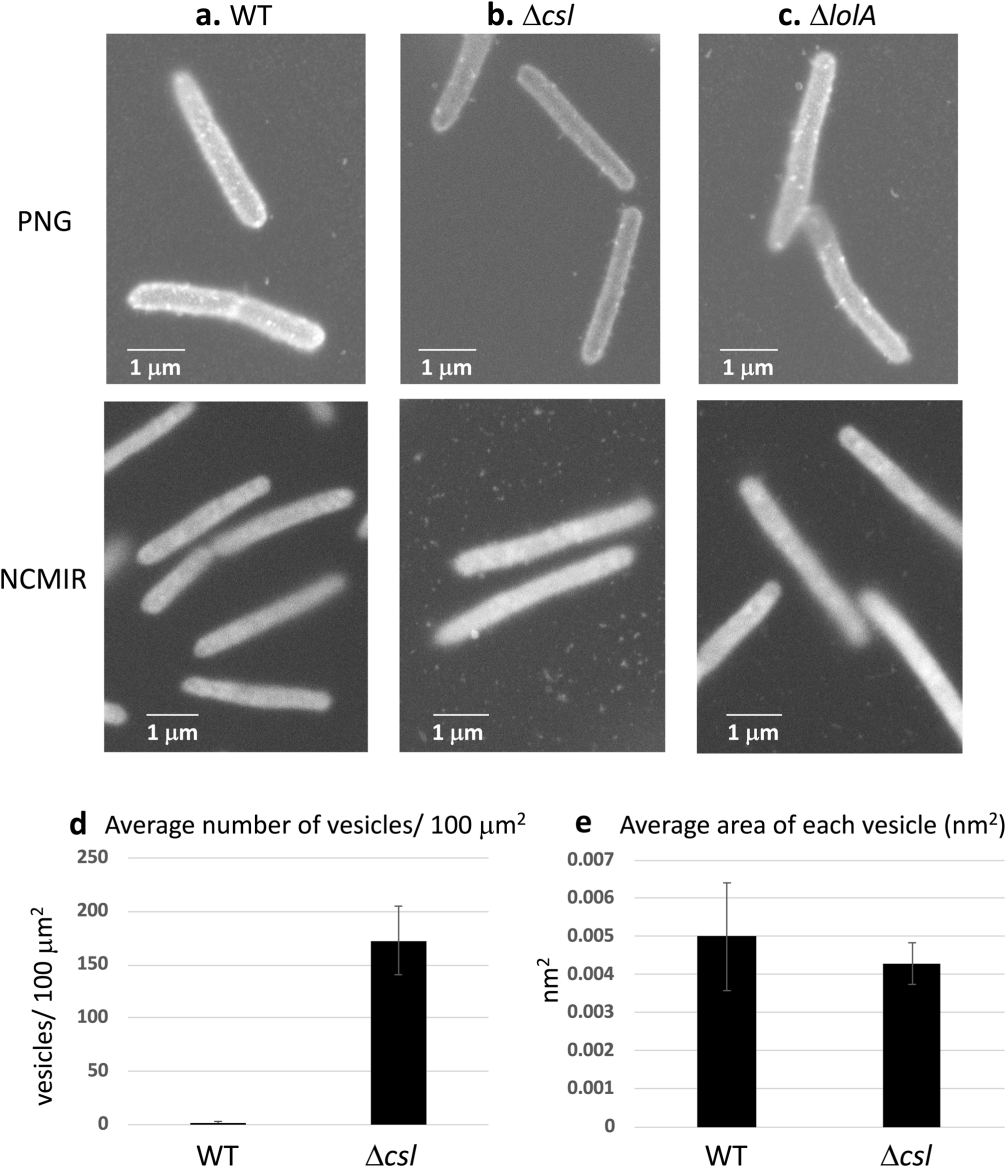
ASEM images of *F. johnsoniae*. Cells were cultured in CYE (casitone-yeast extract) liquid medium directly on the SiN film of an ASEM dish, fixed with paraformaldehyde and glutaraldehyde, and stained as described in the methods section^14^. The cells were immersed in glucose solution and observed using ASEM. (**a**) WT, (**b**) Δ*csl*, (**c**) Δ*lolA*. Upper panels, positively charged Nanogold-labeled cells; lower panels, Nanogold-labeled cells counterstained by the NCMIR method to visualize vesicles. Both the outer membranes and filaments 200 nm in length were clearly imaged for all of the cells. Scale bar 1 μm. (**d**) The polulation density of vesicles (vesicles/100 μm^2^) was measured. More vesicles were released from *Δcsl* cells than from WT cells (Fig. S8). (**e**) The area of vesicles (nm^2^) was measured. Vesicles released from *Δcsl* cells were almost the same size as vesicles released by the WT. The error bars on the graph correspond to the SD.

**Figure 8:**
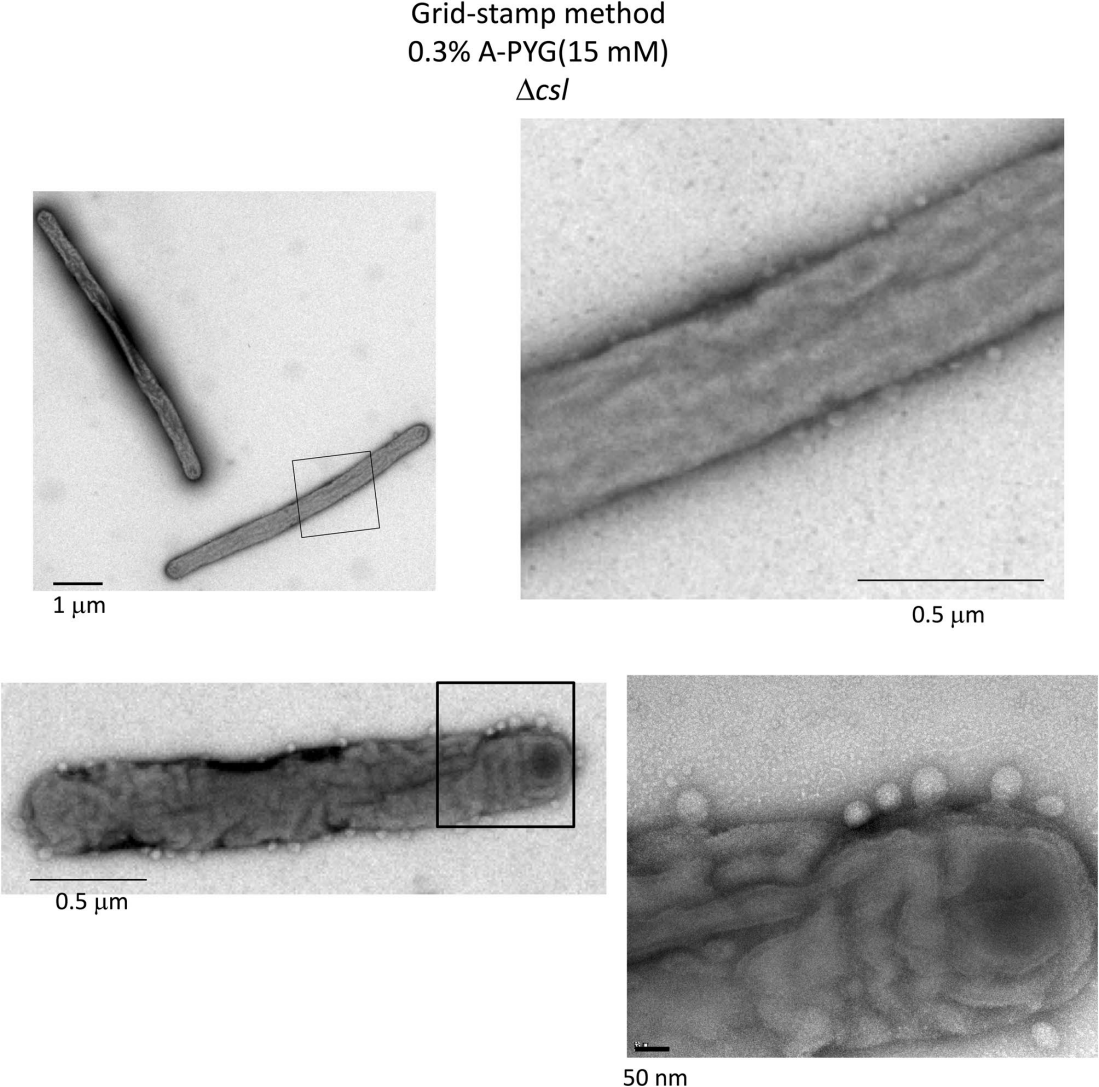
Grid stamp images of the edge of *Δcsl* mutant *F. johnsoniae* spreading colonies on 0.3% A-PYG (15 mM). Left: grid-stamp images of bacteria in the spreading front region. Right: high magnification image of the squares indicated in the left panels. Many vesicles were released from the *Δcsl* cells. The dark spots of high electron density observed in the WT cells on 1% A-PY2^28^ were not observed in *Δcsl* cells on 0.3% A-PYG (15 mM).

To image bacteria in aqueous solution, WT, Δ*sprB*, *gldK*, Δ*lolA* and Δ*csl* cells were dissociated from the respective colonies and cultured individually in liquid medium in ASEM dishes overnight. The cells were then aldehyde-fixed and labeled with positively charged Nanogold particles^29,30^. For each strain, the cells on the SiN film windows were imaged in situ*,* immersed in aqueous liquid, by ASEM. Positively charged Nanogold labeling was possible because Gram-negative bacteria, including *F. johnsoniae,* are surrounded by an outer membrane comprising phospholipids and lipopolysaccharides, which presumably have a negatively charged core oligosaccharide region; the labeling was previously demonstrated for *Escherichia coli* cells^29,30^. Both the outer membranes and almost 200 nm-long filaments protruding from the mutant and WT *F. johnsoniae* cells were clearly labeled, although the released vesicles and the surrounding structures were not clearly observed (Fig. 7a–c, upper). To enhance the lipids and membranes, the cultured cells were stained by a modified NCMIR method using OsO_4_. More membrane vesicles were released from Δ*lolA* and Δ*csl* cells than from WT, Δ*sprB* and *gldK* cells (Fig. 7a–c, lower, Fig. S8). On average, there were 172.7 vesicles/100 μm^2^ and 10.06 vesicles/cell for the Δ*csl* mutant, which is far more than that for the WT strain (1.1 vesicles/100 μm^2^ and 0.06 vesicles/cell (Fig. 7d). Examples of the images used for the analysis are shown in Fig. 7a,b, lower panels; membrane vesicles protruding from Δ*csl* cells are evident (Fig. 7b, lower). The average area of each vesicle was 4.3 nm^2^/vesicle for the Δ*csl* mutant and 5 nm^2^/vesicle for WT *F. johnsoniae* (Fig. 7e). Thus, although abundant vesicle secretion by each Δ*csl* cell was suggested, the size of the secreted vesicles was still comparable to the size of WT vesicles. The spreading fronts of rigid Δ*csl* colonies and soft WT, Δ*sprB* and Δ*lolA* colonies that formed on 0.3% A-PYG (15 mM) were imaged using the Grid Stamp TEM. This allowed the instant when vesicles were secreted from cells in the spreading colonies on 0.3% A-PYG (15 mM) to be documented (Fig. 8). Further, colonies cultured similarly on 0.3% A-PYG (15 mM) were fixed, embedded in Epon, thin-sectioned and imaged by TEM. The images indicate that the outer membranes of WT, Δ*sprB*, Δ*lolA* and Δ*csl* cells were smooth along the long axis of the cell (Fig. S7); the undulations observed along the long axis of cells on 1% A-PY2 were absent (Fig. S7, S9,^23^). The dark spots of high electron density observed in the WT cells on 1% A-PY2 during SprB-dependent colony spreading^28^ were not observed in Δ*csl*, Δ*sprB* and WT cells on 0.3% A-PYG (15 mM) during the same stage (Fig. 8).

## Discussion

Attachment to a solid surface and cell–cell interactions involve complex processes that are affected by the growth medium, substrate, and cell surface. Therefore, morphological assays of bacterial colonies growing on agar media involve the analysis of bacterial motility and chemotaxis, which are influenced by nutrient and agar concentrations. Generally, soft agar migration assays have been widely employed to assess the motility and chemotaxis of *E. coli*, *Helicobacter pylori* and other flagellated bacteria. Previously, we found that colony spreading of *F. johnsoniae* on 1% A-PY2 was dose-dependently inhibited by the addition of glucose^31^. In the present work, SprB-dependent spreading was inhibited by low agar concentrations, suggesting the need for a relatively hard culture substrate.

We have shown that both WT *F. johnsoniae* and the Δ*sprB* mutant form dendritic colonies on 0.3% A-PYG (15 mM) and that these colonies spread during an initial growth-dependent phase and then by a gliding motility-dependent phase; the dendritic pattern of the WT colony was more complex than the dendritic pattern formed by the Δ*sprB* colony (Figs. 2; S1b, S5). We showed that *F. johnsoniae* windmill-like structures, which exhibit periodic stripes, were moved outwards on 0.3% A-PYG (15 mM). Furthermore, we showed that the bacterial cells immediately around and under the windmill-like structures stayed close to them and that the cells in the region surrounding the windmill-like structures made horizontal back-and-forth oscillations (Figs. S2e1-2), that, in total, could result in rotational movement, which could become clearer after a long time period. This movement matched the movement of the windmill-like structure observed by microscopy, suggesting that the oscillations of bacterial cells might cause it. Because windmill-like structures were found in the dendrites of colonies but not in the depressions (Fig. S2e3), the movement of the windmill-like structures is suggested to be involved in dendrite formation or caused by dendrite formation and, further, related to colony expansion. We could not identify what these windmill-like structures were made of, but they were located on the top of the dendrites of the colonies.

On the relatively hard, nutrient-poor surface of 1% A-PY2, WT colonies expanded while keeping the circular form of the initial inoculation spot. In contrast, the area covered by Δ*sprB* cells did not increase appreciably after inoculation on this type of media (Fig. 5d). *Salmonella enterica *and* Yersinia enterocolitica* cells swarm on soft agar media supplemented with glucose, but do not swarm in the absence of glucose^32^. Swarming motility is powered by rotating flagella; the production of flagellar and regulatory systems, including quorum sensing and two component systems, is essential for the regulation of swarming^33^. In *F. johnsoniae*, the system regulating colony spreading has not yet been identified and is the subject of future studies. Similarly, the signaling pathways used to adjust the motility and secretion of substances in response to the surrounding environment are still unknown.

The colony spreading of the *F. johnsoniae* mutants deficient in genes encoding the T9SS or the gliding machinery was also suppressed during the gliding motility-dependent phase on 0.3% A-PYG (15 mM) (Fig. S4). Our data suggest that both the T9SS and the gliding machinery are necessary for motility adhesin SprB-independent colony spreading on 0.3% A-PYG (15 mM).

*Myxococcus xanthus* and *F. johnsoniae*, which belong to the phyla Proteobacteria and Bacteroidetes, respectively, serve as model organisms for different types of gliding motility. *M. xanthus* cells move on soft 0.5% agar medium via social motility (S-motility) using a type IV pilus (T4P)-mediated swarming movement^34^. This movement is accompanied by the secretion of exopolysaccharide, which provides an attachment site for T4Ps of surrounding cells. The mechanism of single-cell gliding at the swarm edges is dependent on the adventurous motility (A-motility) system, not the S-motility system powered by polar T4P^35^. The “focal adhesion” model of *M. xanthus* proposes that cell envelope complexes consisting of inner membrane and periplasmic components attached to a proton motive force (PMF)-driven motor, move along a helical track within the cell, and that this movement is associated with the secretion of slime that forms trails at the leading edge of the swarms^36^. There are some similarities between this swarming movement of *M. xanthus* and the gliding movement of *F. johnsoniae*^12^. Both of these soil bacteria are Gram-negative, and their single cells have elongated shapes. As in the *M. xanthus* model, complex machinery causing gliding motion is thought to span the inner and outer membranes of *F. johnsoniae* cells and connect with cell-surface adhesins^37^. The PMF is thought to provide the energy required for cell surface adhesin migration to the outer membrane. However, T4Ps have not been found in *F. johnsoniae,* and the movement of this bacterium on solid surfaces only depends on the Gld machinery, which includes the T9SS. On the other hand, an unknown adhesin, other than SprB, might be involved in colony expansion on soft 0.3% A-PYG (15 mM), because gliding is a form of motility adhesin-dependent movement.

In the current study, some *F. johnsoniae* cells were observed invading the soft agar culture substrate (Fig. 4). This is attributable to the strong physical movement of *F. johnsoniae,* including spreading and gliding.

What molecules are involved in SprB-independent colony spreading? We anticipated that there might be a molecular complex on the cell that binds cell surface proteins to form the gliding machinery. Five lipoproteins were identified from WT colonies spreading on 0.3% A-PYG (15 mM), although the content of these lipoproteins in the membrane fraction was relatively small. Bacterial lipoproteins are generally thought to be anchored to the cell membrane by N-terminally linked fatty acids, suggesting relatively tight binding. However, at least five lipoproteins were released from the outer membrane of *F. johnsoniae* or anchored to the vesicles secreted (Figs. 5a,b, S6). This might facilitate cell-to-cell communication in biofilm. In *M*. *xanthus*, the outer membrane lipoprotein CglC can be transferred from a *cglC* + donor to a Δ*cglC* mutant in a cell–cell contact-dependent manner and stimulate gliding motility in the recipient. This suggests that the surface-exposed lipoproteins that influence gliding motility in *M*. *xanthus* might move on the cell surface of the attached cell and can also affect the cell.

The T9SS and gliding machinery are necessary for both motility adhesin SprB-dependent and SprB-independent colony spreading. Defining the function of their machinery components and their movement during colony spreading are challenges for the future. The difference in colony morphology depends on the mechanism by which the substance is secreted and on how intercellular communication and passive movements such as sliding and darting are involved. In the present study, we shed light on the biofilm-related social physiology of *F. johnsoniae*. The conclusions presented may hold true for other microorganisms with similar characteristics.

## Methods

### Bacterial strain and biofilm cultivation

*Flavobacterium johnsoniae* strains were grown in casitone-yeast extract (CYE) medium at 30 °C (Becton, Dickinson and Co.). The details of the bacterial strains and plasmids used are shown in Table S1^38,39^. For the selection and maintenance of antibiotic-resistant *F. johnsoniae* strains, antibiotics were added to the medium at the following concentrations: streptomycin, 100 μg/ml; erythromycin, 100 μg/ml.

To observe colony spreading, *F. johnsoniae* cells were grown in CYE medium at 27 °C with shaking (175 rpm) overnight. The cells were collected as a pellet by centrifugation at 800 × g for 10 min at 22 °C. The pellet was resuspended in the same volume of washing buffer (10 mM Tris–HCl pH 7.4) by vortexing, and the suspension was centrifuged at 800 × g for 10 min at 22 °C. These steps were repeated twice. The cells were spotted onto peptone yeast (PY2) or peptone yeast glucose (PYG) agar medium (agar: Ina Food Industry Co., Ltd., Japan) in a dish 9 cm in diameter at 23 °C.

*Flavobacterium johnsoniae* gene deletion mutants were constructed essentially according to the method of R. G. Rhodes et al*.*^38^: DNA regions upstream and downstream of a gene were PCR-amplified from the chromosomal DNA of *F. johnsoniae* using pairs of primers (gene-UF-BamHI plus gene-UR-SalI and gene-DF-SalI plus gene-DR-SphI, respectively, where 'U' indicates upstream, 'F' indicates forward, 'D' indicates downstream, and 'R' indicates reverse). The primers used are listed in Table S1. The amplified DNA was cloned into the pGEM-T Easy vector (Promega). The upstream region was double-digested with BamHI and SalI. The downstream DNA was digested with SalI and SphI. Both digested products were ligated with pRR51 that had been digested with BamHI and SphI.

### Time-lapse video films

Time-lapse video films of the edge of the expanding colonies were produced using TIRFM (OLYMPUS, Japan). 1% of the inoculated cells were fluorescently labeled and grown to form colony on an agar-filled plate. The plate was inverted on the specimen stage, and monitored from underneath using an Olympus BX61 microscope. Fluorescent signals were visualized with a phase contrast objective LUCPlanFLN 20 × (OLYMPUS, Japan) and captured with a monochrome CoolSnapHQ digital camera (Photometrics, USA) using MetaMorph software version 6.1 (Molecular Devices, USA). Exposure times were typically 500 ms for GFP (excitation, 490–510 nm; emission, 520– 550 nm). The phase-contrast microscope images and fluorescence images were taken alternately every 15 s, and the images were merged. The images were further analyzed, adjusted, and cropped using MetaMorph software (Molecular Devices, USA).

To observe the overall spreading of the colonies, a fixed-point observation was taken at 30 min intervals using a LAS 2000 camera system (GE Healthcare Life Sciences, USA).

### Fixation

Spreading colonies on agar medium and cultured bacterial cells were fixed with 1% paraformaldehyde (PFA) and 3.5% glutaraldehyde (GA) in 0.1 M phosphate buffer (PB) (pH 7.4) for 30 min at room temperature (RT) for heavy metal staining and charged Nanogold labeling for ASEM. Biofilms were fixed with 4% PFA for 10 min at RT for immunolabeling for optical microscopy. After labeling, colonies were further fixed with 1% GA for 10 min at RT, after which the contrast of the labels/stain was increased by Nanogold labeling enhancement and/or counterstaining with heavy metals as described previously for ASEM^29^. For Epon embedding and TEM, biofilms were fixed with 2.5% GA in PB at RT for 1 h and further with 1% osmic acid (OA) in the same buffer at 4 °C for 1 h.

### Epon embedding and sectioning

Fixed colonies were dehydrated through a gradient series of alcohols at RT and embedded in Epon 812. Ultrathin sections (70 or 400 nm thick) were cut parallel to the colony spreading direction and perpendicular to the agar medium surface. This allowed both spreading across the surface of the agar medium and any penetration into the agar to be monitored. A Leica Ultracut UCT microtome was employed. A series of ultrathin sections were cut at RT and collected on EM grids.

### TEM imaging

Epon sections were mounted on grids, stained with uranyl acetate (UA) and lead citrate (LC) (TAAB Laboratories Equipment, Aldermaston, England) and observed with an H7600 TEM (Hitachi, Tokyo, Japan) at 80 kV.

### Carbon grid stamp TEM method

The glow-discharged EM grid covered by a thin carbon film was lightly placed on the agar medium, removed again, washed with double distilled water (DDW), stained with 2% UA, and observed with a JEM1230 TEM (JEOL, Tokyo, Japan) at 100 kV.

### NCMIR staining method

Spreading colonies on agar medium and bacterial cells cultured on the SiN film window of ASEM dishes were stained using a slightly modified NCMIR method^29^ described in^30^. Briefly, the fixed colonies were washed with 0.15 M cacodylate buffer containing 2 mM calcium chloride (pH 7.4), further fixed using cacodylate buffer containing 2 mM CaCl_2_, and further fixed/stained with the same buffer supplemented with 1.5% potassium ferricyanide (Sigma-Aldrich, St. Louis, MO, USA) and 2% aqueous osmium tetroxide (OsO_4_) (Nisshin EM) at RT for 20 min. After washing with DDW, tissues were incubated with filtered 1% thiocarbohydrazide (TCH; Tokyo Chemical Industry, Co., Ltd., Tokyo, Japan) at RT for 20 min, rinsed with DDW, further stained with 2% aqueous OsO_4_ at RT for 30 min, rinsed with DDW, stained with 2% UA in DDW and kept at 4 °C overnight. Finally, after rinsing with DDW, the tissue samples were stained with 0.4% LC at RT for 2 min.

### Labeling with charged nanogold

For charged Nanogold labeling, aldehyde-fixed bacteria on an ASEM dish were incubated with a 6 μM solution of positively charged 1.4 nm Nanogold particles (Nanoprobes, Stony Brook, NY, USA) for 20 min at RT^30^. After washing with DDW^29^, the size of the gold particles was increased by gold enhancement using GoldEnhance-EM (Nanoprobes) for 10 min at RT, followed by washing with DDW^29^. The bacteria were imaged in situ by ASEM as described^30,36^.

### ASEM imaging

ASEM images were recorded using the ClairScope ASEM system (JASM-6200), JEOL, Ltd., Tokyo, Japan. ASEM dishes with eight windows^35,40^ were employed. Bacteria were grown directly in casitone-yeast extract (CYE) medium on an ASEM dish overnight; the fixation and staining required for imaging were performed in situ^30^. Colony biofilms were cultured on 0.3% agar plates as described above, fixed as described above, and transferred to an ASEM dish for observation by inverted SEM of the ASEM^36,41^. To image the top of the colony biofilm, after fixation, a colony on the agar layer was excised (5 mm × 5 mm) from the agar plate and stained as described. The block was inverted and placed on the SiN film forming the window of the ASEM dish so that the top of the biofilm was in direct contact with the SiN film. The biofilm was immersed in radical scavenger glucose solution^42^ and immediately imaged by optical microscopy and SEM^43^. The electron dose at the highest magnification of 20,000 × was 2.5 e^-^/Å^2^, which is 5.3% of the dose of 47 e^-^/Å^2^ permitted in low-dose cryo-electron microscopy aiming at atomic resolution single particle reconstructions.

### Surface protein analysis of bacteria

The bacteria of spreading colonies on 0.3% A-PYG (15 mM) were collected, suspended in 10 mM Tris–HCl pH 7.5 and vortexed. The supernatant was collected by centrifugation at 20,000 × g for 10 min at 4 °C. The proteins were precipitated with 10% trichloroacetic acid at 4 °C and harvested by centrifugation at 20,000 × g for 10 min at 4 °C. The pellet was washed 3 times with cold diethyl ether and subjected to SDS-PAGE, followed by mass spectroscopy (MS).

### MS analysis and database search for protein identification

Proteins were identified by peptide mass fingerprinting after in-gel tryptic digestion as described previously^7^.

## Supplementary information

Supplementary Information 1.

Supplementary Table S1.

Supplementary Information S1.

Supplementary Information S2a.

Supplementary Information S2b.

Supplementary Information S2c.

Supplementary Information S2d1.

Supplementary Information S2d2.

Supplementary Information S2d3.

Supplementary Information S2e1.

Supplementary Information S2e2.

Supplementary Information S2e3.

Supplementary Information S3

Supplementary Information S4.

Supplementary Information S5.

Supplementary Information S6.

Supplementary Information S7.

Supplementary Information S8.

Supplementary Information S9.
