# Supplementary material for: Colony spreading of the gliding bacterium Flavobacterium johnsoniae in the absence of the motility adhesin SprB
Source: Sci Rep. 2021 Jan 13;11:967. doi: 10.1038/s41598-020-79762-5 (PMC7807042; doi:10.1038/s41598-020-79762-5)
Supplement: Supplementary file 1 — Supplementary Information 1. [file 41598_2020_79762_MOESM1_ESM.docx]

**Colony spreading of the gliding bacterium *Flavobacterium johnsoniae* in the absence of the motility adhesin SprB**

**Running title: Colony spreading in the absence of Δ*sprB***

Keiko Sato^1*^, Masami Naya ^2^, Yuri Hatano^2^, Yoshio Kondo^3^, Mari Sato^2^, Yuka Narita^4^, Keiji Nagano^5^, Mariko Naito^1^, Koji Nakayama^1^ and Chikara Sato^2*^

^1^Department of Microbiology and Oral Infection, Nagasaki University Graduate School of Biomedical Sciences, Nagasaki, Nagasaki 852-8588, Japan.

^2^ Biomedical Research Institute, Advanced Industrial Science and Technology (AIST), Tsukuba, Ibaraki 305-8566, Japan

^3^ Department of Pediatric Dentistry, Graduate School of Biomedical Sciences, Nagasaki University, 1-7-1 Sakamoto, Nagasaki 852-8588, Japan

^4^ Department of Functional Bioscience, Infection Biology, Fukuoka Dental College, Matsudo, Tamura, Sawara, Fukuoka 814-0913, Japan.

^5^ Department of Microbiology, Health Sciences University of Hokkaido, 1757 Kanazawa, Tobetsu-cho, Ishikari-gun, Hokkaido 061-0293, Japan

**Supplementary Figure Legends**

**Fig. S1. Colony spreading of *F. johnsoniae* on media containing different concentrations of agar and glucose.** **(a)** Optical microscopy image of the colony spreading of WT, Δ*sprB* and Δ*csl* cells in 9-cm diameter dishes (5 days). Panel 1: 1% A-PY2, Panel 2: 0.5% A-PY2, Panel 3: 0.3% A-PY2, Panel 4: 1% A-PYG(5 mM) containing 5 mM glucose, Panel 5: 0.5% A-PYG(5 mM) containing 5 mM glucose, and Panel 6: 0.3% A-PYG(5 mM) containing 5 mM glucose. In the absence of glucose, colony spreading on agar medium decreases as the agar concentration decreases. In the presence of glucose, colony spreading increases as the concentration of agar decreases. **(b)** Colony spreading of WT and Δ*sprB* cells on 1% A-PYG(15mM) (top), 0.5% A-PYG(15mM) (middle), and 0.3% A-PYG(15mM) (bottom). In the presence of 15 mM glucose, WT and Δ*sprB* cells spread well on 0.3% A-PYG(15mM) and formed dendritic colonies.

**Fig. S2. Effect of agar and glucose concentrations on the behavior of WT *F. johnsoniae* colony spreading monitored using Optical microscopy.** Cells expressing GFP in their cytoplasm were added to the inoculated bacterial solution at a concentration of 1% **(a-d)** or 100% **(e)**, and movement of bacterial cells at the colony edge was monitored. Images were recorded by fluorescence microscopy at 30-s intervals for 30 min. Time is shown at the top left. **(a)** WT cells of the spreading colony formed on 1% A-PY2. At the tip of an outer branch of the spreading colony, small cell clusters moved outwards, followed by other cell clusters. **(b)** WT cells in the non-spreading colony formed on 1% A-PYG(15 mM). An enlarged movie of the square in **b1** is shown in the subpanel on the left **(b2)**. In the edge region of the colony, long lines of static cells expressing GFP were visible. The lines can be interpreted as divided cells that remain connected. **(c)** WT cells in the non-spreading colony formed on 0.3% A-PY2. An enlarged movie of the square in **c1** is shown in the subpanel on the left **(c2)**. In the edge region of the colony, most bacterial cells moved back and forth from the same place. **(d1-d3)** Two different dendrite regions and a concave region between two dendrites of a WT colony formed on 0.3% A-PYG(15 mM). The dendrite tips examined are indicated by red circles in the upper low magnification image **(d1, d2)**. Bacterial cells moved in all directions, and windmill-like structures with crystal-like stripes were sometimes clearly imaged on the dendritic surface **(d1, d2)**. The concave region examined is indicated by a red circle **(d3)**; neither windmill-like nor crystal-like structures were apparent. **(e)** The dynamics of bacterial cells around a windmill-like structure. The movement of the bacterial cells was monitored a round windmill-like structures formed near the edge surface of a colony by 100% GFP- expressing cells. **(e1)** *Upper left panel*; Phase contrast image of the edge of a dendrite tip. A windmill-like structure was found on the surface. *Upper right panel*; Fluorescence movie of the surface of the region monitored using CLSM. *Lower left panel*; Montage of the phase contrast and the first image of the movie. Cells were distributed around the windmill-like structure, and oriented similarly for each arm. *Lower right panel*; Color representation of the motion of cells in the ‘*upper right’* movie. **(e2)** Arrow representation of cell track superimposed on the lower right panel in **e1**. The movements of each cell~~s~~ are somewhat specific and related to the orientation of their neighboring ‘windmill’ arm, and, overall, the group including the windmill-like structure rotated counterclockwise, and shifted toward the bottom left of the imaged region. **(e3)** *Upper left panel*; the same as the upper left panel in **e1**. *Upper right panel*; stack representing the 3D cell distribution determined using CLSM. *Lower left panel*; the top surface image of the *upper right stack.* The cell distribution is related to windmill-like structure. *Lower middle panel*; the middle image of the *stack. Lower right panel*; the bottom image of the *stack.* The cell distribution is not related to the windmill-like structure.

**Fig. S3. Two- step colony spreading of WT and Δ*sprB* on 0.3% A-PYG(15 mM) monitored using Optical microscopy.** Images were recorded at 60-min intervals using a LAS3000 (Luminescent image analyzer system). An initial growth-dependent phase is followed by a secondary gliding motility-dependent phase. Time is indicated at the top left.

**Fig. S4. Effects of various mutations related to T9SS or motility on the colony spreading of *F. johnsoniae* on 0.3% A-PYG(15 mM).** The colony spreading of *gld* and *spr* mutants were compared with that of WT *F. johnsoniae*. Spreading was much reduced by all of the mutations, and only some mutants showed signs of dendrite formation. The gliding machinery, including T9SS, has an essential role for the colony spreading.

**Fig. S5. Colony spreading of WT, Δ*lolA*, Δ*sprB* and double mutant Δ*lolA*, Δ*sprB* cells.** **(a)** Colony spreading of WT and Δ*lolA* cells on 1% A-PY2 (left) and 0.3% A-PYG(15 mM) (right). **(b)** Colony spreading of Δ*sprB* and Δ*sprB* Δ*lolA* cells on 1% A-PY2 (left) and 0.3% A-PYG(15 mM) (right). Neither the colonies formed by Δ*lolA* nor the colonies formed by Δ*sprB* Δ*lolA* spread on 1% A-PY2 or 0.3% A-PYG(15 mM).

**Fig. S6. Comparison of the membrane proteins and soluble proteins of WT, Δ*lolA*, and Δ*csl* colonies formed on 0.3% A-PYG(15 mM).** The SDS- PAGE patterns of membrane and cytoplasmic fractions from WT, Δ*lolA*, and Δ*csl* cells were similar, except for the protein band arising from Fjoh_3856 (arrow).

**Fig. S7. The surfaces and membranes of cells in colonies formed on 0.3% A-PYG imaged by TEM.** Washed cells of WT, Δ*sprB*, Δ*lolA*, and Δ*csl* were inoculated on 0.3% A-PYG and incubated for 4 days. The colonies formed were aldehyde-fixed and embedded in Epon. Afterwards, 70 nm thin sections were cut along the long cell axis of the cell. The outer membranes of WT, Δ*sprB*, Δ*lolA* and Δ*csl* cells were smooth along the long axis of the cell.

**Fig. S8. ASEM images of *F. johnsoniae*.** Cells were cultured in CYE (Casitone-yeast extract) liquid medium directly on the SiN film of an ASEM dish, fixed with paraformaldehyde and glutaraldehyde, and stained as described in the methods section (14, 23). The cells were immersed in glucose solution and observed using ASEM. **(a)** Δ*sprB*, **(b)** *gldK*. Left panels, positively charged Nanogold labelling; right panels, Nanogold labeled cells counter-stained by the NCMIR method to visualize vesicles. Both the outer membranes and filaments 200 nm in length were clearly imaged for all the cells. Scale bar 1 μm.

**Fig. S9. The surfaces and membranes of cells in colonies formed on 1% A-PY2 imaged by TEM.** Washed WT, Δ*sprB*, Δ*lolA*, and Δ*csl* were inoculated and cultured on 1% A-PY2 and the colonies formed were fixed and embedded in Epon, then 70 nm thin sections sampling the cells were cut. **(a)** Sections cut along the long axis of the cell. **(b)** Sections cut along the short axis of the cell. The membranes of WT (23: Fig. S5 left), Δ*sprB* (23: Fig. S5 right), Δ*lolA* and Δ*csl* *F. johnsoniae* only displayed undulations along the long cell axis.
