# Supplementary material for: Colony spreading of the gliding bacterium Flavobacterium johnsoniae in the absence of the motility adhesin SprB
Source: Sci Rep. 2021 Jan 13;11:967. doi: 10.1038/s41598-020-79762-5 (PMC7807042; doi:10.1038/s41598-020-79762-5)
Supplement: Supplementary file 13 — Supplementary Information S3 [file 41598_2020_79762_MOESM13_ESM.pptx]

## Slide 1
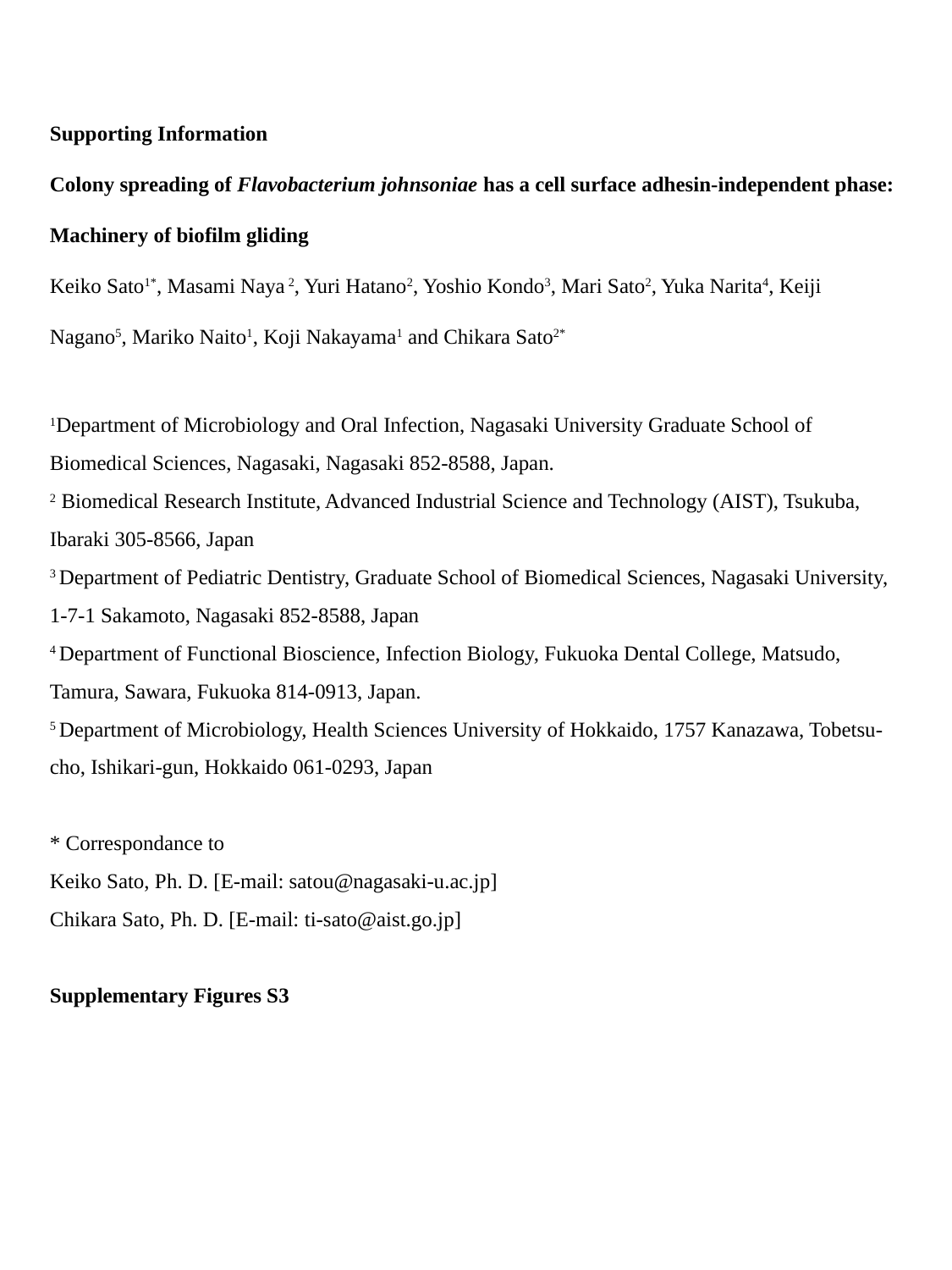

Supporting Information
Colony spreading of Flavobacterium johnsoniae has a cell surface adhesin-independent phase: Machinery of biofilm gliding
Keiko Sato1*, Masami Naya 2, Yuri Hatano2, Yoshio Kondo3, Mari Sato2, Yuka Narita4, Keiji Nagano5, Mariko Naito1, Koji Nakayama1 and Chikara Sato2*
1Department of Microbiology and Oral Infection, Nagasaki University Graduate School of Biomedical Sciences, Nagasaki, Nagasaki 852-8588, Japan.
2 Biomedical Research Institute, Advanced Industrial Science and Technology (AIST), Tsukuba, Ibaraki 305-8566, Japan
3 Department of Pediatric Dentistry, Graduate School of Biomedical Sciences, Nagasaki University, 1-7-1 Sakamoto, Nagasaki 852-8588, Japan
4 Department of Functional Bioscience, Infection Biology, Fukuoka Dental College, Matsudo, Tamura, Sawara, Fukuoka 814-0913, Japan.
5 Department of Microbiology, Health Sciences University of Hokkaido, 1757 Kanazawa, Tobetsu-cho, Ishikari-gun, Hokkaido 061-0293, Japan
* Correspondance to
Keiko Sato, Ph. D. [E-mail: satou@nagasaki-u.ac.jp]
Chikara Sato, Ph. D. [E-mail: ti-sato@aist.go.jp]
Supplementary Figures S3

## Slide 2
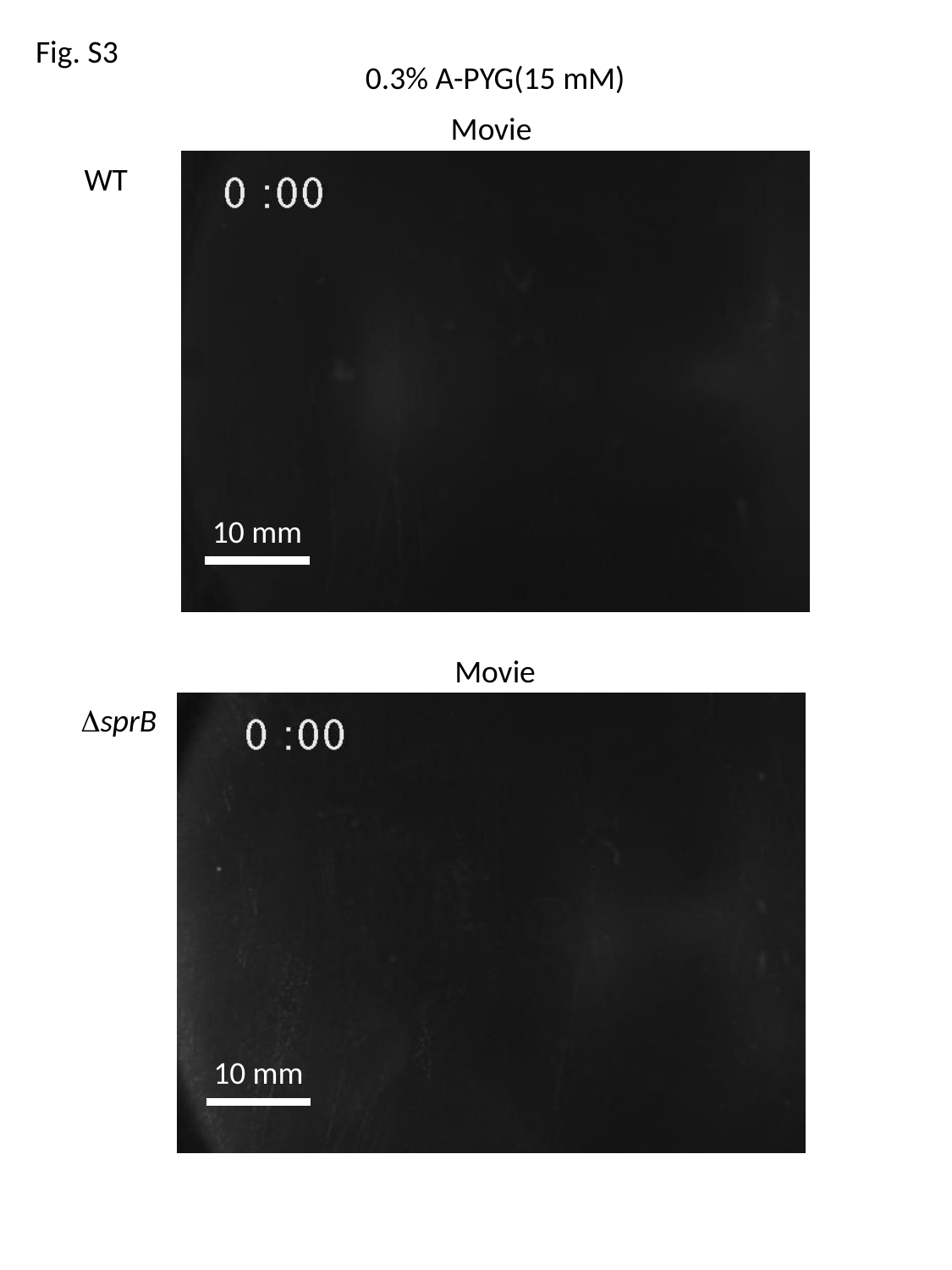

Fig. S3
0.3% A-PYG(15 mM)
Movie
10 mm
WT
10 mm
DsprB
Movie
